# Cross-population variation in usage of a call combination: evidence of signal usage flexibility in wild bonobos

**DOI:** 10.1007/s10071-024-01884-4

**Published:** 2024-08-30

**Authors:** Isaac Schamberg, Martin Surbeck, Simon W. Townsend

**Affiliations:** 1 Dept. of Evolutionary Anthropology, University of Zürich, Zürich, Switzerland; 2 Center for the Interdisciplinary Study of Language Evolution (ISLE), University of Zürich, Zürich, Switzerland; 3 Dept. of Human Evolutionary Biology, Harvard University, Cambridge, MA USA; 4 School of Anthropology and Museum Ethnography, Oxford University, Oxford, UK; 5 Dept. of Human Behavior, Ecology and Culture, Max Planck Institute for Evol. Anthropology, Leipzig, Germany; 6 Dept. of Psychology, University of Warwick, Warwick, UK

**Keywords:** Arbitrariness, Bonobos, Animal communication, Animal signals, Evolution of language, Call combinations, Primate communication

## Abstract

The arbitrary relationship between signifier and signified is one of the features responsible for language’s extreme lability, adaptability, and expressiveness. Understanding this arbitrariness and its emergence is essential in any account of the evolution of language. To shed light on the phylogeny of the phenomenon, comparative data examining the relationship between signal form and function in the communication systems of non-humans is central. Here we report the results of a study on the production and usage the whistle-high hoot call combination (W + HH) from two distant populations of wild bonobos (*Pan paniscus*): Lui Kotale, DRC, and Kokolopori, DRC. We find that the context in which bonobos produce the W + HHs varies systematically between populations. Our results suggest that variation in W + HH production may represent an example of signal-adjustment optionality, a key component of arbitrariness.

**Supplementary Information:**

The online version contains supplementary material available at 10.1007/s10071-024-01884-4.

## Introduction

The relationship between a signal’s form and its function is foundational to all systems of communication. The nature of the association profoundly influences the expressive potential of a communication system (Hockett 1960). In language, the relationship between a word’s sound and its meaning is said to be ‘arbitrary’ because in most cases, the association is a matter of socio-linguistic convention, rather than an obligatory or natural connection (Saussure 1916). Such arbitrariness is one of the key design features responsible for language’s extreme lability and adaptability (Gasser 2004). Understanding arbitrariness, and its evolution, therefore, is essential in any account of the evolution of language. To shed light on the phylogeny of the phenomenon, it is necessary to take a comparative approach and examine arbitrariness (and related capacities) in the communication of non-human animals.

No non-human communication system appears to exhibit the degree of arbitrariness present in language, but the precise connection between signal and function (or meaning) in animal communication remains an open question. Several studies have challenged the notion that arbitrariness is unique to language by, for example, documenting changes in call structure across time. Chimpanzees, for example, appear to be able to modify the acoustic structure of their long-distance calls in response to social influences at the dyadic (Mitani and Gros-Louis 1998) and group level (Crockford et al. 2004). They have also been shown to be able to learn to modify their food calls when exposed to conspecifics who produce a different call variant (Watson et al. 2015).

The extent to which these examples of sound change, or ‘signal-adjustment optionality’ (*sensu* Watson et al. 2022), are mirrored by a similar capacity for ‘semantic change’, or ‘signal-usage optionality’ (*sensu* Watson et al. 2022), is largely unknown. Both pig-tailed macaques and Japanese macaques have been shown to be able to be trained by humans to use ‘coo’ calls to request particular items from humans, demonstrating the ability to use an existing signal in service of a novel function (Coudé et al. 2011; Hihara et al. 2003). These examples suggest that the association between a signal and its function is not immutable. However, the extent to which this potential for ‘signal-usage optionality’ occurs in the natural communication of non-humans is largely unknown.

We address this question by comparing usage of a complex signal, the whistle-high hoot call combination (W + HH), produced in two populations of wild bonobos separated by 455k (the Kokolopori and LuiKotale field sites). Previous work has demonstrated the call combination is an antiphonal signal used during interparty communication (Schamberg et al. 2016, 2017). In the current study we present data on the contexts in which W + HHs are produced in order to investigate potential shifts in call usage across populations.

## Methods

### Study sites and subjects

**Fig. 1:**
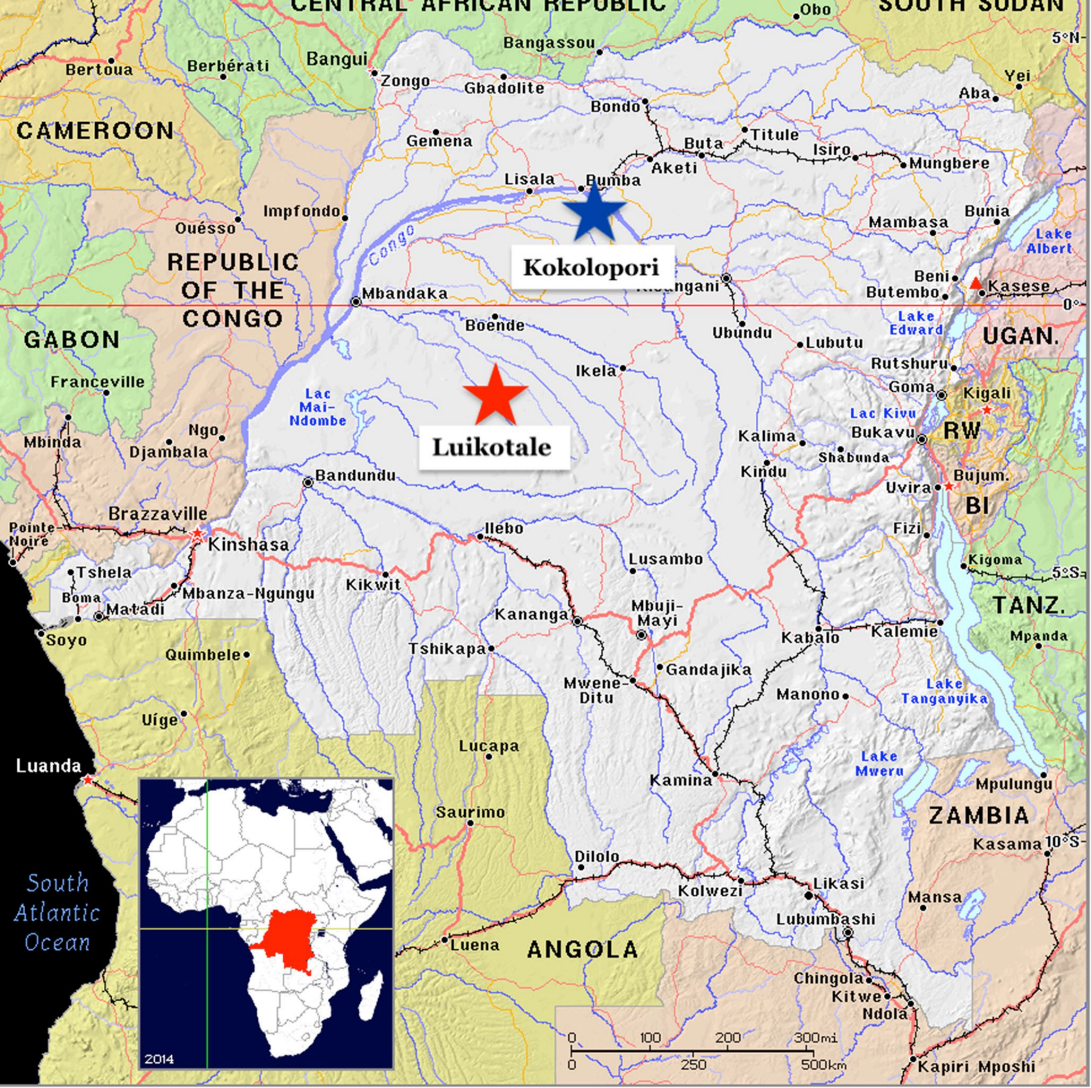
The locations of the Kokolopori and Luikotale field sites on a map of the Democratic Republic of Congo

Data for this study were collected at two field sites: LuiKotale (Hohmann and Fruth 2003) and Kokolopori (Surbeck et al. 2017). Home ranges for both communities were located in dense rainforest consisting of large patches of both terra firma and swamp forest. The two field sites are separated by 455 km. Several impassable rivers render any migration or contact between the bonobos at the two field sites impossible (see Fig. 1). Details of each field site are given below.

### LuiKotale field site

For 13 months between July 2011 and March 2014, Isaac Schamberg (IS) sampled behavior and recorded vocalizations from 18 adults (7 males and 11 females, aged 10 + years) from a single group of bonobos (the Bompusa community) at the LuiKotale field site in the Mai-Ndombe province of the Democratic Republic of Congo (DRC). Individuals in this community have been studied continuously since 2002 and were fully habituated and identified at the beginning of the study.

### Kokolopori field site

From January-May 2018, IS sampled behavior and recorded vocalizations from two groups of bonobos (the Ekalakala and Kokoalongo communities) at the Kokolopori field site in the Equateur, DRC. The Ekalakala community consisted of 3 adult males, and 6 adult females. The Kokoalongo community consisted of 10 adult males, and 17 adult females. Bonobos in these communities have been studied continuously since 2016 and were fully habituated from the beginning of the data collection period. Given the small number of individuals in the Ekalakala community and the similar patterns observed across the two Kokolopori communities, we combined data from the Ekalakala and Kokoalongo communities into a single Kokolopori dataset (but see SI for data from each individual community).

### Data collection and processing

Subjects were followed on foot and observations were conducted between 0600 and 1800. IS recorded vocalizations and accompanying behavior with an audio recorder (Marantz PMD 660; Sennheiser directional microphone) and later transcribed the recordings. Data were collected over the course of 1515 observation hours (1224 h at LuiKotale and 291 h at Kokolopori).

When an individual produced a vocalization, the observer noted the call type, the caller’s behavior, the identity of individuals within 10 m of the caller, immediate behavioral change after the call, and all vocalizations produced by the caller and by other individuals that preceded or followed the call. Initial classification of call types in the field were later confirmed through visual inspection of spectrograms using the criteria laid out in published accounts of the bonobo vocal repertoire (de Waal 1988; Bermejo and Omedes 1999). We also evaluated the reliability of our categorization of call types by conducting a test of inter-rater reliability, in which a second coder, who was naïve to the hypotheses of the current study, classified 171 call units from 25 recordings from our dataset (representing 10% of the total dataset). The two coders agreed on 95% (161/171) of categorizations of ‘whistles’ and ‘high hoots’ (Cohen’s kappa = 0.91), indicating high degree of reliability in call categorization.

We classified each call combination in the dataset into one of four contexts, which were defined as follows:

Travel: calls produced while the caller was walking terrestrially were classified in the ‘travel’ context. If an individual paused his/her travel for less than one minute, the utterance was still considered t0 have been produced in a travel context.

Arrival: calls produced two minutes before or after arriving at a feeding patch were classified in the ‘arrival’ context. Call production typically occurred near the base of a fruiting tree, or in a fruiting tree prior to feeding.

Feeding: calls produced while the caller was actively feeding were classified in the ‘feeding’ context. If an individual paused his/her feeding for less than one minute, the utterance was still considered t0 have been produced in a feeding context.

Rest: calls produced while callers were stationary (but not currently feeding) were classified in the ‘rest’ context.

### Statistical analysis

Statistical analysis was conducted in R 3.6.1 GUI 1.70 El Capitan build. Although our sample size was somewhat constrained, we still opted for using GLMMs since our dataset exceeded minimum thresholds (*N* = 25) proposed in recent meta-analyses examining the use GLMMs with limited datapoints (Jenkins and Quintana-Ascencio 2020).

## Relationship between population and W + HH context

To investigate potential differences in the usage of W + HH call combinations between the Luikotale and Kokolopori populations, we fitted a generalized linear mixed model with a poisson error structure and log link function using the function ‘glmer’ in R package ‘lme4’ (lme4 (version 1.1–27.1). The response variable was the number of W + HH call combinations each individual produced in the four contexts. Each individual, therefore, is represented by four data points (one for each call context). We included the following predictor variables: population (LuiKotale/Kokolopori), context (arrival/feeding/rest/travel), the observation time of each individual, and the interaction between population and context. We included observation time as a predictor variable to control for variation in observation time between individuals. Individual identity was entered as a random effect.

Because W + HHs are produced somewhat rarely (Schamberg et al. 2016), we included all observed W + HHs in our dataset.

## Relationship between population and HH context

To investigate whether any between-population differences in W + HH production were specific to the call combination, or simply reflective of more general pattern of behavioral or vocal change across communities, we also examined usage of HH between the LuiKotale and Kokolopori populations. We classified the production context of each HH bout using the same criteria we used for classifying the W + HH combinations. We fitted the same generalized linear mixed model described above, but the response variable was the number of HHs–instead of W + HHs– produced by each individual in each context. If HH and W + HH usage co-vary across the two populations, such variation can be explained as part of a broader change in behavior. Conversely, if HH and W + HH usage do not co-vary across the two populations, variation in either one of the signals–HHs or W + HHs–would likely be evidence of a signal-specific difference between the populations.

For Kokolopori, all high-quality recordings of HHs produced by individuals who also produced at least one W + HH were included in our dataset. HHs from LuiKotale came from the same dataset as was used in Schamberg et al. (2016).

## Results

### Whistle-high hoots

**Fig. 2:**
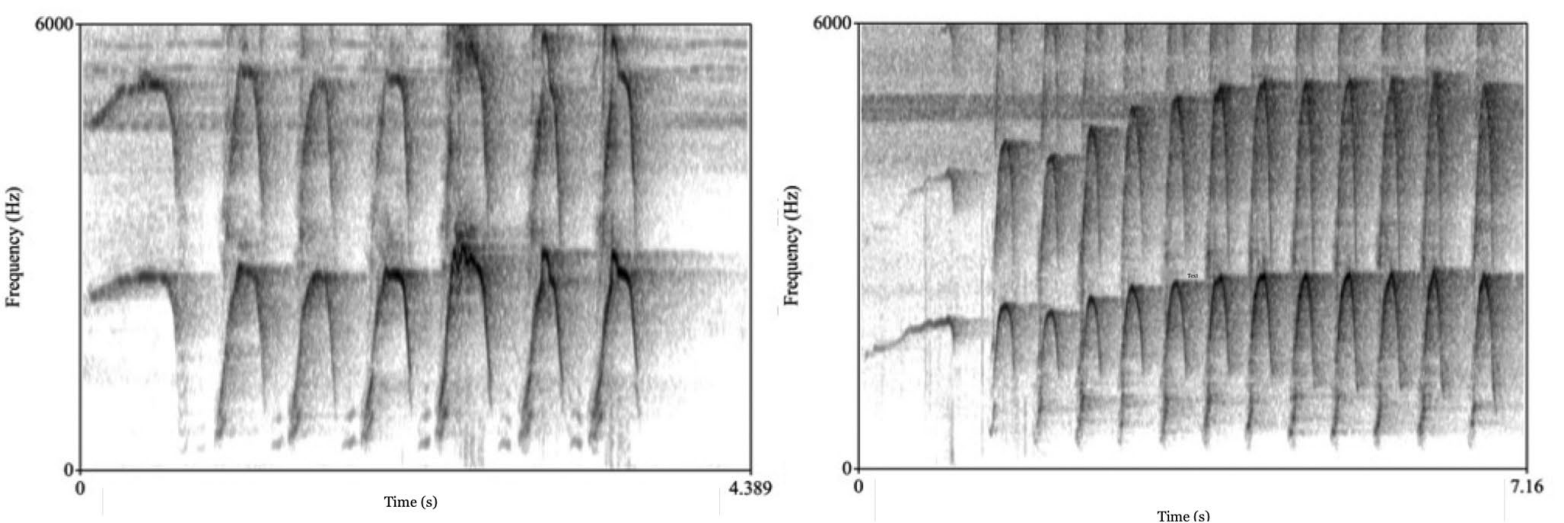
Spectograms depicting whistle-high hoots (W + HHs) produced the Kokolopori field site (left) and the LuiKotale field site (right)

At the Kokolopori field site, we recorded 42 W + HH combinations from 15 individuals (8 males, 7 females). At the LuiKotale field site, we recorded 51 W + HH combinations from 14 individuals (7 males, 7 females). At both field sites, call order was invariant: 100% of W + HH combinations consisted of an initial *whistle*, followed by one or more *high hoots* (see Fig. 2).

**Fig. 3:**
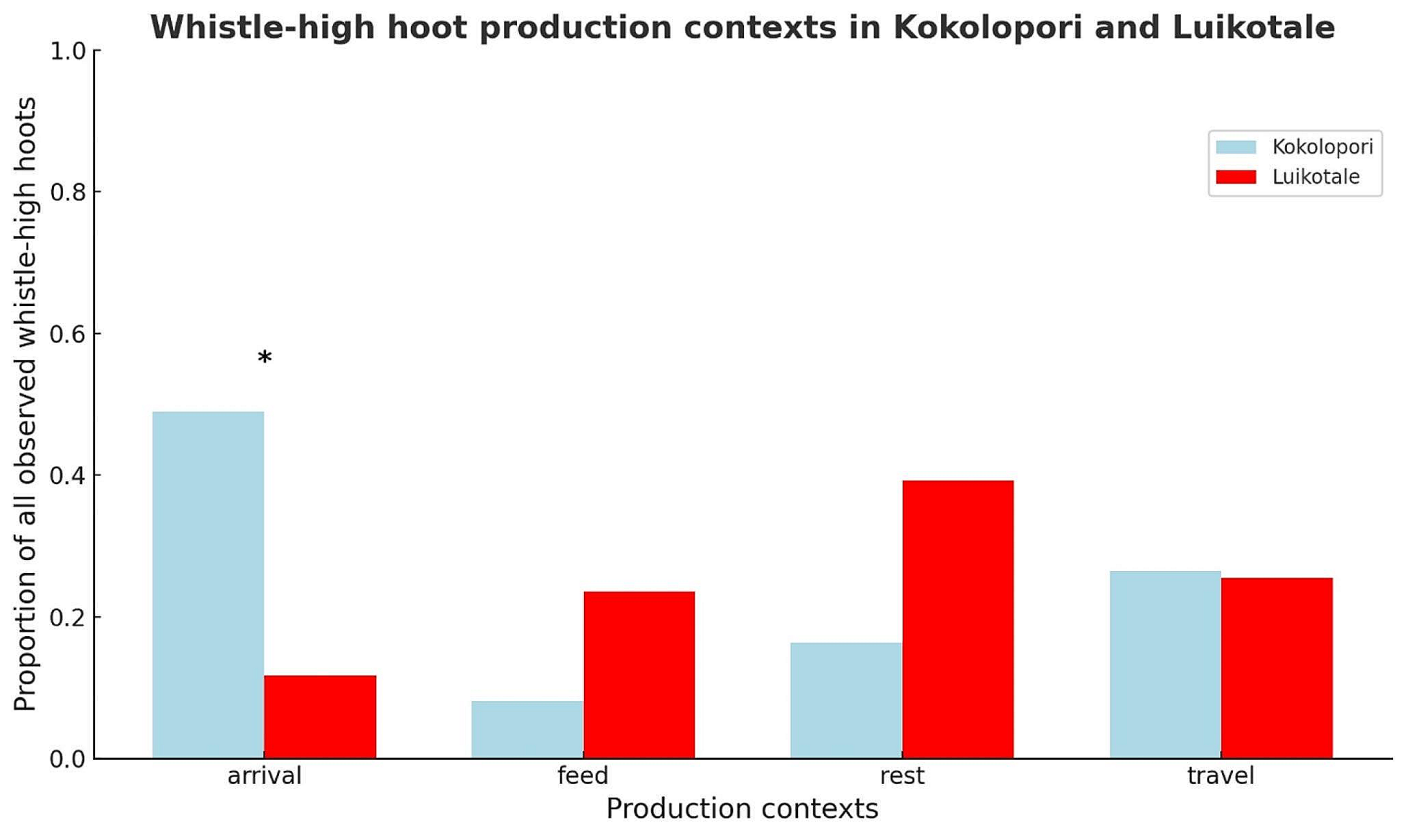
The proportion of whistle-high hoot (W + HH) call combinations produced in each of four contexts (arrival, feed, rest, and travel) among two populations of bonobos

At both sites, bonobos produced W + HHs in all four contexts, but the predominant context accompanying call production differed between the two populations (Fig. 3). At Kokolopori, the majority (22/42) of W + HHs were produced upon arrival at a fruiting tree. At LuiKotale, a plurality (20/52) of W + HHs were produced while resting. Comparison of the null model (which included ‘‘population’ and ‘context’ as predictor variables) and the full model (which included ‘population’, ‘context’, and an the ‘population*context’ interaction) was significant (df = 3, χ^2^ = 20.67, *p* < 0.001), indicating that there was a difference in the context of call production between the two populations. Results from Model 1 suggest that this difference may be driven by a high proportion of W + HHs produced in the arrival context by bonobos at Kokolopori (β=-2.7, SE = 0.8, z=-3.4, *p* = 0.007) (See S1 for full results of Model 1).

### High hoots

At both sites, bonobos produced a clear majority of high hoots during periods of feeding or resting (75/95 at LuiKotale and 37/51 at Kokolopori). Comparison of the null model (which included ‘‘population’ and ‘context’ as predictor variables) and the full model (which included ‘population’, ‘context’, and an the ‘population*context’ interaction) was not significant (df = 3, χ2 = 4.311, *p* = 0.230), indicating no difference in the production of HHs between the two populations.

## Discussion

Our results reveal a cross-population difference in bonobos’ use of the whistle-high hoot (W + HH) call combination. Bonobos at the Kokolopori field site were significantly more likely to produce W + HHs upon arrival at a feeding tree, compared to bonobos at the LuiKotale field site. In contrast, we found no difference in the usage of high hoots (HHs) between the two populations. The contrasting results of HHs and W + HHs indicate that the shift in W + HH usage observed between LuiKotale and Kokolopori does not reflect a broader change in activity budgets or a general tendency to vocalize in particular contexts. Rather, it suggests that the difference in W + HH usage is potentially an example of signal-usage optionality with individuals in the two populations using the W + HH combination for subtly different purposes. This variation in the context of call production–and the possible accompanying change in signal meaning–provides, to our knowledge, some of the first evidence of a capacity for a signal usage adjustment among wild primates.

Within the existing literature on flexibility in primate call usage, there are numerous examples of individuals modifying their vocal output as a function of context and/or social knowledge. For example, individuals may decide whether or not to call based on the identity of nearby individuals (e.g., Townsend et al. 2008; Kalan and Boesch 2015; Soldati et al. 2022), their relationship with a receiver (Silk et al. 2016), the direction of a receiver’s gaze’ Schel et al. 2013), and even the knowledge state of receivers (Crockford et al. 2012). These examples demonstrate strategic, volitional call usage by individual callers.

Our results extend these findings by not only providing further evidence for volitional call production, but also demonstrating a group-wide pattern of convergent call usage. Such group-conforming call usage appears similar to social traditions that have been identified in other behavioral domains [e.g., Perry et al. 2003; Whiten et al. 2001). To our knowledge, our findings provide the first preliminary evidence for a vocal tradition based on the manner in which a particular signal is used by a group of primates (but see Wich et al. 2012 for a vocal tradition based on call selection; and Crockford et al. 2004; Ford 1991; Yurk et al. 2002, and Weilgart and Whitehead 1997 for vocal traditions based on convergent acoustics). The capacity for subtly shifting the precise contexts of production of vocalization–may represent an important stepping stone towards the fully-fledged arbitrariness of human language. Comparing vocal usage across great ape communities and populations is essential to shed light on how widespread this phenomenon is and, as a consequence, how deeply rooted it may be within the primate lineage.

As a result of the current study’s specific scope, our results raise more questions than they answer. We would like to highlight two perspectives that we hope future research will address. First, we documented a difference in W + HH usage between LuiKotale and Kokolopori, but our data do not allow for inferences about a possible concomitant difference in signal meaning across the two populations. Our results are equally consistent with two potential interpretations: (1) LuiKotale and Kokolopori bonobos produce W + HHs in different contexts because the meaning of the signal differs between the two populations; or (2) LuiKotale and Kokolopori bonobos produce W + HHs in different contexts, but the meaning of the signal is identical in both populations. A playback experiment probing receiver behavior in the two populations would help disentangle these competing hypotheses regarding signal meaning.

Second, there is good evidence that while primates are largely born with a fixed vocal repertoire, they must *learn* how to correctly use those hardwired call types (e.g., Wegdell et al. 2019). If learning really does play an important role in shaping how primate call usage, between-group differences in call usage should be common. To our knowledge, such differences have rarely been examined, and would be a fruitful direction for future research.

## Electronic supplementary material

Below is the link to the electronic supplementary material.

Supplementary Material 1

## Data Availability

Data is available in the supplementary information.

## References

[CR01] Bermejo M, Omedes A (1999) Preliminary vocal repertoire and vocal communication of wild bonobos (Pan paniscus) at Lilungu (Democratic Republic of Congo). Folia Primatologica 70(6):328–357 10.1159/000021717 10640882

[CR1] Coudé G, Ferrari PF, Rodà F, Maranesi M, Borelli E, Veroni V, Monti F, Rozzi S, Fogassi L (2011) Neurons controlling voluntary vocalization in the macaque ventral premotor cortex. PLoS ONE 6(11):e26822. 10.1371/journal.pone.0026822 22073201 10.1371/journal.pone.0026822 PMC3206851

[CR2] Crockford C, Herbinger I, Vigilant L, Boesch C (2004) Wild chimpanzees produce group-specific calls: a case for vocal learning? Ethology 110(3):221–243. 10.1111/j.1439-0310.2004.00986.x

[CR3] Crockford C, Wittig RM, Mundry R, Zuberbühler K (2012) Wild chimpanzees inform ignorant group members of danger. Curr Biol 22(2):142–146. 10.1016/j.cub.2011.11.053 22209531 10.1016/j.cub.2011.11.053

[CR4] de Saussure F (1916) Nature of the linguistic sign. Course Gen Linguistics 1:65–70

[CR05] de Waal FB (1988) The communicative repertoire of captive bonobos (Pan paniscus), compared to that of chimpanzees. Behaviour 106(3–4):183–251

[CR5] Ford JK (1991) Vocal traditions among resident killer whales (Orcinus orca) in coastal waters of British Columbia. Can J Zool 69(6):1454–1483. 10.1139/z91-202

[CR6] Gasser M (2004) The origins of arbitrariness in language. In Proceedings of the annual meeting of the Cognitive Science Society (Vol. 26, No. 26)

[CR8] Hihara S, Yamada H, Iriki A, Okanoya K (2003) Spontaneous vocal differentiation of coo-calls for tools and food in Japanese monkeys. Neurosci Res 45(4):383–389. 10.1016/S0168-0102(02)00240-7 12657451 10.1016/s0168-0102(03)00011-7

[CR9] Hockett C (1960) The origin of speech. Sci Am 203(3):88–97 14402211

[CR10] Hohmann G, Fruth B (2003) Lui Kotal-A new site for field research on bonobos in the Salonga National Park. Pan Afr News 10(2):25–27

[CR11] Jenkins DG, Quintana-Ascencio PF (2020) A solution to minimum sample size for regressions. PLoS ONE 15(2):e0229345 32084211 10.1371/journal.pone.0229345 PMC7034864

[CR12] Kalan AK, Boesch C (2015) Audience effects in chimpanzee food calls and their potential for recruiting others. Behav Ecol Sociobiol 69. 10.1007/s00265-015-1967-6

[CR13] Mitani J, Gros-Louis J (1998) Chorusing and call convergence in chimpanzees: tests of three hypotheses. Behaviour 135(8):1041–1064

[CR14] Perry S, Baker M, Fedigan L, GrosLouis J, Jack K, MacKinnon K, Manson J, Panger M, Pyle K, Rose L (2003) Social conventions in wild white-faced capuchin monkeys: evidence for traditions in a neotropical primate. Curr Anthropol 44(2):241–268

[CR15] Schamberg I, Cheney DL, Clay Z, Hohmann G, Seyfarth RM (2016) Call combinations, vocal exchanges, and interparty movement in wild bonobos. Anim Behav 122:109–116. 10.1016/j.anbehav.2016.11.001

[CR16] Schamberg I, Cheney DL, Clay Z, Hohmann G, Seyfarth RM (2017) Bonobos use call combinations to facilitate inter-party travel recruitment. Behav Ecol Sociobiol 71:1–8. 10.1007/s00265-016-2234-5

[CR17] Schel AM, Townsend SW, Machanda Z, Zuberbühler K, Slocombe KE (2013) Chimpanzee alarm call production meets key criteria for intentionality. PLoS ONE 8(10):e76674. 10.1371/journal.pone.0076674 24146908 10.1371/journal.pone.0076674 PMC3797826

[CR019] Silk JB, Seyfarth RM, Cheney DL (2016) Strategic use of affiliative vocalizations by wild female baboons. PLoS One 11(10):e0163978 10.1371/journal.pone.0163978 PMC5081171 27783705

[CR19] Soldati A, Fedurek P, Dezecache G, Call J, Zuberbühler K (2022) Audience sensitivity in chimpanzee display pant hoots. Anim Behav 190:23–40. 10.1016/j.anbehav.2022.03.014

[CR20] Surbeck M, Coxe S, Lokasola AL (2017) Lonoa: the establishment of a permanent field. site for behavioral research on bonobos in the Kokolopori

[CR021] Townsend SW, Deschner T, Zuberbühler K (2008) Female chimpanzees use copulation calls flexibly to prevent social competition. PLoS One 3(6):e2431 10.1371/journal.pone.0002431 PMC3278306 22423311

[CR21] Watson SK, Filippi P, Gasparri L, Falk N, Tamer N, Widmer P, Manser M, Glock HJ (2022) Optionality in animal communication: a novel framework for examining the evolution of arbitrariness. Biol Rev 97(6):2057–2075 35818133 10.1111/brv.12882 PMC9795909

[CR050] Watson SK, Townsend SW, Schel AM, Wilke C, Wallace EK, Cheng L, ... Slocombe KE (2015) Vocal learning in the functionally referential food grunts of chimpanzees. Current Biol 25(4):495–499 10.1016/j.cub.2014.12.032 25660548

[CR026] Wegdell F, Hammerschmidt K, Fischer J (2019) Conserved alarm calls but rapid auditory learning in monkey responses to novel flying objects. Nature Ecol Evol 3(7):1039–1042 10.1038/s41559-019-0903-5 31133723

[CR024] Weilgart L, Whitehead H (1997) Group-specific dialects and geographical variation in coda repertoire in South Pacific sperm whales. Behavior Ecol Sociobiol 40:277–285

[CR022] Whiten A, Goodall J, McGrew WC, Nishida T, Reynolds V, Sugiyama Y, ... Boesch C (2001) Charting cultural variation in chimpanzees. Behaviour 1481–1516

[CR023] Wich SA, Krützen M, Lameira AR, Nater A, Arora N, Bastian ML, ... van Schaik CP (2012) Call cultures in orang-utans?. PLoS one 7(5):e36180 10.1371/journal.pone.0036180 PMC3346723 22586464

[CR025] Yurk H, Barrett-Lennard L, Ford JKB, Matkin CO (2002) Cultural transmission within maternal lineages: vocal clans in resident killer whales in southern Alaska. Animal Behaviour 6(63):1103–1119

