# Supplementary material for: Cross-population variation in usage of a call combination: evidence of signal usage flexibility in wild bonobos
Source: Anim Cogn. 2024 Aug 30;27(1):58. doi: 10.1007/s10071-024-01884-4 (PMC11364580; doi:10.1007/s10071-024-01884-4)
Supplement: Supplementary file 1 — Supplementary Material 1 [file 10071_2024_1884_MOESM1_ESM.docx]

**Supplementary Information**

Table S1. Full results of the generalized linear mixed model

| Model 1 | Production context of whistle-high hoot (W+HH) call combination in two bonobo populations | | | |
| --- | --- | --- | --- | --- |
| Full model | # of W+HH produced ~ (1\| ID) + context+ population + observation time + context * population | | | |
| Null Model | # of W+HH produced ~ (1\| ID) + context+ population + observation time | | | |
| Full-null comparison | df= 3, χ^2^ = 20.67 , p= 0.0001 | | | |
|  | Estimate | SE | z | p |
| (Intercept) | -1.70 | 0.62 | -2.75 | **0.006**** |
| Context (rest) | 0.98 | 0.68 | 1.45 | 0.148 |
| Context (travel) | 1.10 | 0.68 | 1.65 | 0.100 |
| Context (arrival) | 1.99 | 0.62 | 3.24 | **0.001**** |
| Population (LK) | 2.23 | 0.96 | 2.32 | **0.021*** |
| Observation time | -0.0313 | 0.0314 | -0.996 | 0.319 |
| Rest*LK | -0.47 | 0.77 | -0.61 | 0.542 |
| Travel*LK | -1.02 | 0.78 | -1.31 | 0.191 |
| Arrival*LK | -2.68 | 0.79 | -3.39 | **0.007***** |

Table S2. Observations of Whistle-high hoots (W+HH) from the Bompusa community at Luikotale field site

Table S3. Observations of Whistle-high hoots (W+HH) from the Ekalakala community at Kokolopori field site

Table S4. Observations of Whistle-high hoots (W+HH) from the Kokoalongo community at Kokolopori field site
